# How does COVID-19 kill at home and what should we do about it?

**DOI:** 10.1093/eurheartj/ehaa599

**Published:** 2020-09-03

**Authors:** Hanno L Tan

**Affiliations:** e1 Department of Cardiology, Heart Center, Amsterdam University Medical Center location AMC, University of Amsterdam, Meibergdreef 9, 1105 AZ Amsterdam, The Netherlands; e2 Netherlands Heart Institute, Utrecht, The Netherlands

## Abstract

**Figure:**
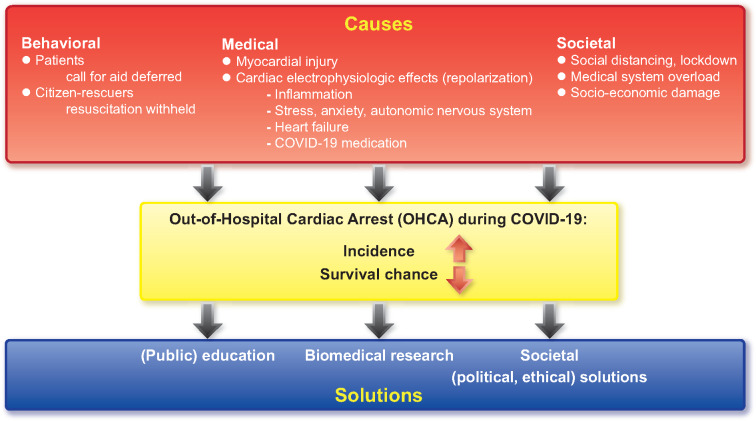

**This editorial refers to ‘COVID-19 kills at home: the close relationship between the epidemic and the increase of out-of-hospital cardiac arrests’^†^, by E. Baldi *et al*., on page 3045.**

The COVID-19 pandemic is causing upheaval across countless areas of medicine and society. The cardiovascular effects of COVID-19 are key impacts of the disease and include myocardial injury, cardiac arrhythmia, arterial and venous thrombo-embolism, and heart failure. Their presence is associated with reduced chances of survival. Also, disease severity is higher, and survival chances lower, in patients with pre-existing heart disease.^1^^,^^2^

Baldi and co-workers now provide, in this issue of the *European Heart Journal*, convincing evidence that COVID-19 is also associated with increased incidence of out-of-hospital cardiac arrest (OHCA).^3^ During the first 2 months of the COVID-19 pandemic in Northern Italy in 2020, the OHCA incidence rose by 52% compared with the corresponding period in 2019, and OHCA victims with confirmed or suspected COVID-19 constituted 74% of excess OHCA cases. The changes in OHCA incidence closely followed those in COVID-19 infections. Also, changes associated with reduced chances of surviving OHCA occurred: longer Emergency Medical Services (EMS) arrival times (12 min in 2019 vs. 15 min in 2020), higher proportions of OHCAs occurring at home and/or unwitnessed and lower proportions at public places and/or witnessed, and lower provision of cardiopulmonary resuscitation (CPR) by citizen-rescuers. Accordingly, the proportion of patients dead on arrival of the EMS was larger, and the proportion of patients transported with return of spontaneous circulation (ROSC) dropped by 57%. The survival rate to hospital discharge declined (from 9.5% to 5.1%), but not statistically significantly, presumably due to limited numbers. Most differences were also found between OHCA victims with confirmed or suspected COVID-19 and non-COVID-19 OHCA victims.

This study is consistent with a similar study conducted simultaneously by Marijon and co-workers in Paris.^4^ Marijon found that OHCA incidence doubled over the first 6 weeks of the COVID-19 pandemic, and patients with confirmed or suspected COVID-19 constituted ∼33% of excess OHCA cases. The same key determinants of survival were less favourable during the pandemic (rise in median EMS response time from 9.4 to 10.4 min, larger proportion of OHCA incidences at home, lower proportions of witnessed OHCA and shockable rhythm), resulting in reduced survival rates to hospital admission (23% to 13%) and hospital discharge (5.4% to 3.1%).

The strength of both studies is that they derive from large registries dedicated to study OHCA in the general population, thereby limiting inclusion bias. One important limitation is, however, that both registries only enrol OHCA patients with EMS involvement. This may have caused an underestimation of the problem, because EMS would be uninvolved in cases where OHCA occurred unwitnessed and the patients were found after they had already died. These patients (who mostly die at home) may contribute to the gap, reported in several countries, between observed excess mortality during the COVID-19 pandemic and death tolls attributed to confirmed or suspected COVID-19 (typically patients dying in hospital).^5^

Still, both studies together provide compelling evidence for an association between COVID-19 and OHCA. Moreover, they indicate that the problem of OHCA, already a major general health problem causing 20% of total mortality and 50% of mortality in patients with known heart disease in industrialized societies in the pre-COVID-19 era, has only worsened during the COVID-19 era.

How can we solve this problem? First, we must understand the mechanisms linking COVID-19 to increased OHCA risk (*Take home figure*). We must look beyond medical–biological reasons alone, as patients with confirmed or suspected COVID-19 infection constitute only 33%^4^ to 74%^3^ of excess OHCA cases.

**Take home figure:**
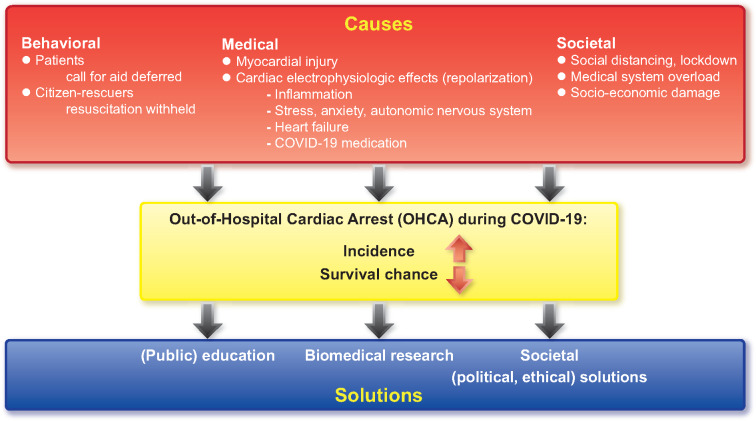
Possible causes of increased incidence of and reduced survival chances after out-of-hospital cardiac arrest during COVID-19 pandemic, and possible management strategies.

Some findings may result from the rapid spread of COVID-19 and the subsequent societal lockdown. The instructions for citizens to maintain social distancing and remain at home can partly explain the lower proportion of witnessed OHCA, and consequently the lower chances of being alive^3^ and/or having shockable rhythm at EMS arrival.^4^ Also, longer EMS response times are consistent with medical system overload at the start of the pandemic. Some observations, however, indicate that actions (not) taken by patients and by potential citizen-rescuers may also have contributed. In particular, Baldi observed that the number of EMS calls with an eventual diagnosis of ST-segment elevation myocardial infarction (STEMI) dropped by a staggering 40%^3^. While this mirrors observations in other countries,^6^ it is unlikely that STEMI incidence had really decreased (the opposite is more probable: stress evoked by major crises increases the incidence of STEMI and OHCA^7^). It is more likely to reflect the patient’s hesitance to seek medical attention (possibly from fear of contracting COVID-19 in the process). Thus, patient behaviour could contribute to the observed increase in OHCA incidence (STEMI is a major trigger of OHCA) and the poorer outcome of OHCA (delayed treatment may have resulted in a more advanced and complex stage of STEMI). Other observations indicate that changed citizen-rescuer behaviour may also have reduced survival chances, in particular the lower provision of CPR^3^ and of public automated external defibrillator use.^4^ These observations could be explained by the lower proportion of OHCA in public places, but also by more reluctance among citizen-rescuers to provide close physical aid through fear of contracting COVID-19.

Recognizing such behavioural changes is important, because they require appropriate corrective actions, e.g. through public campaigns in which citizens are advised to not defer a call for medical assistance in case of acute complaints, and bystanders to perform CPR as they would in the pre-COVID-19 era. Accordingly, CPR guidelines of the European Resuscitation Council were adapted to reduce the risk of contracting COVID-19 while providing CPR, and include instructions both for professionals and for citizen-rescuers. These actions are crucial to regain the increases in survival rates after OHCA that were painstakingly gained in the pre-COVID-19 era thanks to an intensive and successful partnership between professionals and citizen-rescuers.^8^

What biological reasons underlie increased OHCA risk during COVID-19? Myocardial injury probably causes many cardiovascular effects of COVID-19, and may result from viral myocarditis, stress cardiomyopathy, and/or other systemic consequences of COVID-19 infection, e.g. sepsis and disseminated intravascular coagulation. Myocardial injury may explain increased occurrence of malignant arrhythmias and OHCA, e.g. reentrant arrhythmias supported by myocardial scars. Indeed, elevated biomarker levels of injury in hospitalized COVID-19 patients are associated with higher risk of mortality^2^ and of malignant ventricular tachyarrhythmias.^1^ Yet, half of hospitalized patients with malignant arrhythmias have no myocardial injury, suggesting that other factors with direct impact on cardiac electrophysiology enhance their arrhythmia risk. Several mechanisms are plausible, and all converge on blocking of cardiac potassium channels which largely drive cardiac repolarization. First, inflammatory cytokines [interleukin-1 (IL-1), IL-6, and tumour necrosis factor α (TNFα)] may modulate the function and/or expression of these channels, leading to QT prolongation and increasing the risk of malignant ventricular arrhythmias and OHCA.^9^ Clinical indications that inflammatory mediators invoked by viral infection may, in general, cause malignant arrhythmia are an increased incidence of such arrhythmias in carriers of implantable cardioverter defibrillators (ICDs) during high influenza activity.^10^ Moreover, IL-6 inhibits cytochrome P450, which metabolizes various QT-prolonging drugs.^9^ This is relevant because some such drugs have been considered as COVID-19 treatments, e.g. chloroquine and azithromycin. Indeed, potentially hazardous QT prolongation was observed in hospitalized patients receiving these drugs.^11^ Also, QT prolongation occurs during heart failure secondary to down-regulation of various potassium channels. Finally, QT prolongation may result from psychosocial stress and anxiety, and lead to increased OHCA risk. This is mediated by dysregulation of cardiac ion channels by the autonomic nervous system, in particular, through sympathovagal imbalance.^12^

Despite the many plausible causes of QT prolongation and malignant ventricular tachyarrhythmias during COVID-19, most malignant cardiac arrhythmias in hospitalized COVID-19 patients are bradyarrhythmias and unrelated to QT prolongation, but probably secondary to metabolic derangements caused by advanced COVID-19, e.g. hypoxaemia.^13^ This, however, does not rule out the possibility that QT prolongation is a trigger for malignant arrhythmias in out-of-hospital COVID-19 patients with less advanced COVID-19. Still, observations by Baldi provide no support for this notion:^3^ QTc values in post-ROSC ECGs of OHCA victims were only mildly prolonged (median QTc was 464 ms, while QTc is considered hazardous if >500 ms), and the proportions of OHCA victims with QTc prolongation were identical among COVID-19 patients and non-COVID-19 patients (67%). However, the number of OHCA patients with analysable post-ROSC ECGs was small (*n* = 18, including six with COVID-19). Clearly, larger numbers are required; these could be compiled in consortia dedicated to OHCA research, e.g. the ESCAPE-NET consortium.^14^

Still, the ball may not even stop here. The societal and economic havoc that COVID-19 has already wrought and that is projected to continue for years to come may both increase the OHCA incidence and decrease the chances of surviving OHCA, if it impairs the socio-economic position of large areas of society, because both phenomena are strongly linked to low socio-economic position.^15^

Clearly, our concerted efforts to combat the COVID-19 crisis must also focus on OHCA, and include efforts both to understand the causes that underlie increased OHCA mortality associated with COVID-19, and to implement actions to reduce OHCA incidence and increase chances of surviving OHCA. This may feel like an uphill battle with medical, behavioural, and socio-economic effects of COVID-19 stacked against us. Fortunately, we experienced from the significant increases in OHCA survival rates during the pre-COVID-19 era which resulted from the dogged pursuit of both drastic and incremental changes in resuscitation strategies that these efforts are sure to pay off.

## Footnotes

^†^ doi: 10.1093/eurheartj/ehaa508.

## Notes

The opinions expressed in this article are not necessarily those of the Editors of the *European Heart Journal* or of the European Society of Cardiology.
